# The role of high serum triglyceride levels on pancreatic necrosis development and related complications

**DOI:** 10.1186/s12876-023-02684-9

**Published:** 2023-02-24

**Authors:** Nils Jimmy Hidalgo, Elizabeth Pando, Piero Alberti, Rodrigo Mata, Nair Fernandes, Montse Adell, Sara Villasante, Laia Blanco, Joaquim Balsells, Ramon Charco

**Affiliations:** 1 Universitat Autonoma de Barcelona, Bellaterra, Spain; 2 Department of Hepato-Pancreato-Biliary and Transplant Surgery, Hospital Universitari Vall d’Hebron, 119 Passeig de la Vall d’Hebron, 08035 Barcelona, Spain

**Keywords:** Acute pancreatitis, Triglyceride, Pancreatic necrosis, Hypertriglyceridemia

## Abstract

**Background:**

The relevance of elevated serum triglyceride (TG) levels in the early stages of acute pancreatitis (AP) not induced by hypertriglyceridemia (HTG) remains unclear. Our study aims to determine the role of elevated serum TG levels at admission in developing pancreatic necrosis.

**Methods:**

We analyzed the clinical data collected prospectively from patients with AP. According to TG levels measured in the first 24 h after admission, we stratified patients into four groups: Normal TG (< 150 mg/dL), Borderline-high TG (150–199 mg/dL), High TG (200–499 mg/dL) and Very high TG (≥ 500 mg/dL). We analyzed the association of TG levels and other risk factors with the development of pancreatic necrosis.

**Results:**

A total of 211 patients were included. In the Normal TG group: 122, in Borderline-high TG group: 38, in High TG group: 44, and in Very high TG group: 7. Pancreatic necrosis developed in 29.5% of the patients in the Normal TG group, 26.3% in the Borderline-high TG group, 52.3% in the High TG group, and 85.7% in the Very high TG group. The trend analysis observed a significant association between higher TG levels and pancreatic necrosis (*p* = 0.001). A multivariable analysis using logistic regression showed that elevated TG levels ≥ 200 mg/dL (High TG and Very high TG groups) were independently associated with pancreatic necrosis (OR: 3.27, 95% CI − 6.27, *p* < 0.001).

**Conclusions:**

An elevated TG level at admission ≥ 200 mg/dl is independently associated with the development of pancreatic necrosis. The incidence of pancreatic necrosis increases proportionally with the severity of HTG.

**Supplementary Information:**

The online version contains supplementary material available at 10.1186/s12876-023-02684-9.

## Introduction

Acute pancreatitis (AP) is a highly prevalent disease associated with local (necrosis, abscesses, and pseudocysts) and systemic complications such as persistent single or multisystemic organ failure [1–3]. It is widely described that pancreatic necrosis is one of the worst complications during severe acute pancreatitis, with mortality rates up to 35% [4–6]. In that line, early identification of patients at increased risk of pancreatic necrosis is crucial to initiate interventions such as aggressive fluid resuscitation, organ failure prevention, infection prevention, or earlier admission to an intensive care unit [7–9].

Hypertriglyceridemia (HTG) is a known etiology of acute pancreatitis. However, the exact mechanism of pathophysiology is not clearly defined. The most accepted theory is that the excess triglycerides are hydrolyzed by pancreatic lipase, forming high concentrations of free fatty acids [10]. Free fatty acid and micelle complexes damage the pancreas's vascular endothelium and acinar cells. The resulting ischemia creates an acidic environment leading to the release and activation of pancreatic lipase and proteases, leading to increased autodigestion [11]. Excessed free fatty acids also cause β-cell dysfunction due to impaired mitochondrial function [12]. Collateral injury to pancreatic β-cell can lead to type 3c diabetes and loss of insulin secretion [13, 14]. Some studies also suggest that diabetes increases the severity of AP [15], and insulin protects acinar cells from cellular injury [16, 17].

The HTG is commonly present in the early stage of non-HTG-induced AP, and its clinical significance remains unclear. Some studies have found that triglyceride (TG) elevation upon admission of patients with AP predicts poor prognosis and local and systemic complications [18–22].

However, no studies specifically analyze the association between HTG and the occurrence of pancreatic necrosis in patients with non-HTG-induced AP. Considering that pancreatic necrosis does not necessarily imply organ failure and that non-necrotic pancreatitis can be accompanied by organ failure [23], a specific analysis of the role of TG in patients with pancreatic necrosis becomes necessary.

The HTG in the early phases of acute pancreatitis has been explained due to systemic lipolysis secondary to acute inflammation and the release of pancreatic lipases [24]. The mechanism proposed for pancreatic necrosis development in HTG are the impairment in microvascular circulation due to increased viscosity and direct damage to pancreatic cells mediated by TG degradation products (free fatty acids) [11, 25]. Therefore, the release of TG could have an important role in developing or worsening pancreatic necrosis and be helpful as an early marker.

Our study aimed to ascertain the role of high stratified serum TG levels at admission in developing pancreatic necrosis and its related complications.

## Methods

### Study design

A prospective single-cohort observational study of adult patients diagnosed with acute pancreatitis in a third-level referral center was designed to evaluate the role of high stratified serum TG in developing pancreatic necrosis.

### Study population

#### Inclusion criteria

(1) Patients aged over 18 years with the diagnosis of AP, (2) determination of TG levels at admission (first 24 h), and (3) performing contrast computed tomography (CT) during hospitalization.

AP was defined according to the revised Atlanta Classification 2012 [26]. The diagnosis of AP requires two of the following three features: (a) typical radiating abdominal pain, (b) serum amylase or lipase more than three times normal values, and (c) radiological findings suggestive of pancreatitis on contrast computed tomography (CT), magnetic resonance imaging, or abdominal ultrasound studies.

#### Exclusion criteria

(1) Patients with AP of HTG etiology: AP of HTG etiology was defined as when serum TG levels on admission were ≥ 1000 mg/dL or 500–1000 mg/dL accompanied by lactescent serum in the absence of another etiology of pancreatitis [27–29], (2) patients with coexistence of another major complication whose origin is not AP (gastrointestinal bleeding, duodenal perforation, bile duct perforation), (3) other etiology not related to AP (periampullary neoplasia or of the biliary tract of the proximal or middle third, severe infectious pathology), (4) transferred patients, and (5) patients who arrived at the emergency department with more than 72 h after the onset of symptoms to reduce the bias of including patients with prolonged disease.

### Management of AP

Management of AP patients was done according to international guidelines: initial fluid therapy was installed according to patient characteristics (ringer lactate, physiological sodium solution) for a urinary output of ≥ 0.5 ml/kg/hr. No empirical use of ATB. The patient was referred to an intensive care unit for management when severe AP was suspected [30].

### Triglycerides determination and classification

We measured the serum TG levels in the first 24 h of admission to avoid alterations in TG values that can occur due to factors such as prolonged fasting or administration of parenteral nutrition. TG levels were measured by enzymatic techniques based on spectrophotometric methods (Beckman Coulter Method). In our laboratory, the normal reference intervals are 43–200 mg/dL, regardless of the sex and age of the patient.

We classified patients according to the triglyceridemia stratification proposed by the National Cholesterol Education Program-Adult Treatment Panel III (NCEP-ATP III) [31] and were divided into four groups: Normal TG (< 150 mg/dL), Borderline-high TG (150–199 mg/dL), High TG (200–499 mg/dL) y Very high TG (≥ 500 mg/dL).

### Data collection

The clinical-demographic data collected included age, sex, and body mass index (BMI). We recorded previous diabetes mellitus, arterial hypertension, cardiovascular disease, chronic lung disease, pre-existing chronic kidney disease, and dyslipidemia. The etiology of AP was classified as biliary, alcoholic, idiopathic, post-ERCP, and others (drugs, pancreas divisum, autoimmune, intraductal papillary mucinous neoplasm, post-surgical procedure).

### Computed tomography and pancreatic necrosis

The evaluation of AP with computed tomography was performed. All procedures have a portal venous phase 35 s after administering intravenous contrast. CT was performed at least 24 h after the onset of abdominal pain and preferably between 72 and 96 h. The indications for performing CT in our hospital were: suspicion of moderate/severe or severe AP, presence of persistent SIRS, differential diagnosis with other causes of acute abdomen, and etiological study of non-biliary AP.

*Pancreatic necrosis* was defined as the absence of enhancement in pancreatic tissue after contrast-enhanced CT. Infected pancreatic necrosis (IPN) was defined as a positive culture for microorganisms after necrosectomy or interventional drainage (radiological or endoscopic) [32].

### Local complications

The local complications evaluated were fluid collections, pancreatic necrosis, pancreatic necrosis infection, and the need for invasive procedures against necrosis (radiological, endoscopic, or surgical).

We also evaluated the radiological severity of AP using the classical CT severity index classification [33] and the modified CT severity index [34, 35].

### Systemic complications and outcomes

We assessed the severity of AP based on the 2012 revision of the Atlanta Classification [26]. Mild AP is characterized by the absence of local or systemic complications, while the presence of persistent organ failure defines severe AP. The moderately severe category includes transient organ failure, patients with deterioration of pre-existing comorbidities, and patients with local complications on imaging. Organ failure was defined using the Modify Marshall scoring system [36] as a score of 2 or more for one of three organs (renal, cardiovascular, or respiratory). Persistent organ failure was defined as any organ failure for more than 48 h.

Mortality was defined as a death that occurred during admission or up to 90 days after discharge.

### Other biochemical markers at admission

Laboratory markers analyzed were creatinine, hematocrit, blood urea nitrogen (BUN), and C-reactive protein. Based on thresholds established in previous studies, the following values were considered elevated: creatinine ≥ 1.8 mg/dL [37], hematocrit ≥ 44% [38], BUN ≥ 20 mg/dL [39], C-reactive protein ≥ 15 mg/dL [40].

### Statistical analysis

Chi-square test or Fisher's exact test were used to analyze qualitative variables. Quantitative variables were analyzed using the Kruskal–Wallis test, and the qualitative variables using linear-by-linear association. For normal distributions, the quantitative variables were compared by Student's t-test for two groups, and the nonparametric test used was the Mann–Whitney U test. The Cochran-Armitage trend test was used to evaluate the presence of a statistically significant trend association between TG levels and pancreatic necrosis categories. Multivariable logistic regression analysis was performed to analyze risk factors associated with pancreatic necrosis. Receiver-operating characteristic (ROC) curves for pancreatic necrosis and the area under the curve (AUC) were calculated using TG levels, biochemical markers, and scoring systems. A value of *p* < 0.05 was considered statistically significant. We performed the statistical analyzes using IBM SPSS software, version 20.0 (IBM Corp. in Armonk, NY) and Stata version 16 (Stata, College Station, Texas, USA).

### Ethics

This study was performed in line with the principles of the Declaration of Helsinki. Approval was granted by the Ethics Committee of Hospital Universitari Vall d'Hebron (PR-AG 328/2017). All participants signed informed consent to participate in our prospective register.

## Results

**Fig. 1:**
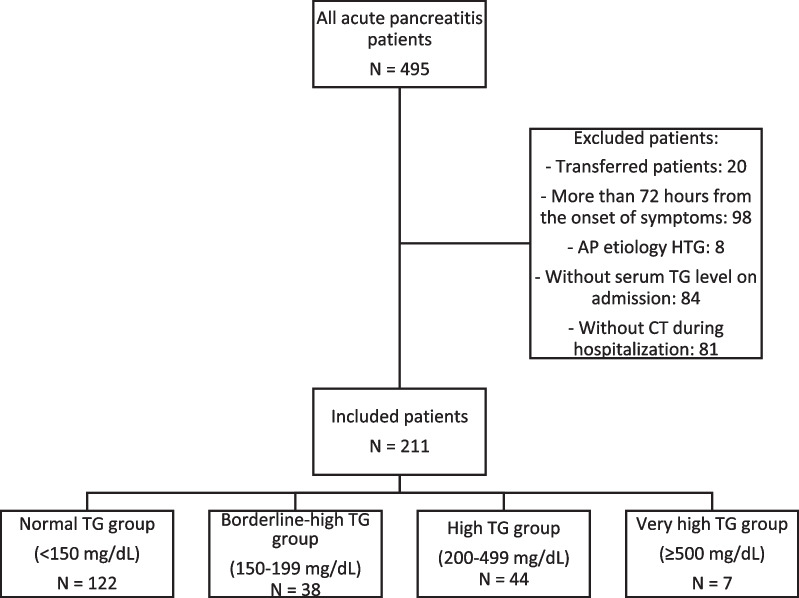
Patient enrollment according to inclusion and exclusion criteria. AP: acute pancreatitis, TG: triglyceride, HTG: hypertriglyceridemia

Between January 2016 and August 2021, after inclusion and exclusion criteria, 211 patients were included. (Fig. 1). After applying the exclusion criteria of having a CT scan, we did not find differences in baseline characteristics when performing an intermediate analysis between the entire initial cohort and the final population. No statistically significant differences were observed between patients with CT and without CT in mean TG levels (167.5 ± 127 mg/dL vs. 129.9 ± 54.7 mg/dL, *p* = 0.137).

Patients were divided into four groups according to the TG level at admission: 122 patients in the Normal TG group (< 150 mg/dL), 38 in the Borderline-high TG group (150–199 mg/dL), 44 in the High TG group (200–499 mg/dL) and 7 in the Very high TG group (≥ 500 mg/dL). No differences were found among groups except in age and pancreatitis etiology (Table 1). The mean TG (mg/dL) was 96.95 ± 27.18 in the Normal TG group, 166.21 ± 11.82 in the Borderline-high group, 278.75 ± 69.06 in the High TG group, and 705 ± 160.48 in the Very high group.

**Table 1:** Demographic and clinical characteristics of acute pancreatitis according to TG levels group

	Normal TG (< 150 mg/dL) N = 122	Borderline-high TG (150–199 mg/dL) N = 38	High TG (200–499 mg/dL) N = 44	Very high TG (≥ 500 mg/dL) N = 7	*p* value
Age, mean ± SD	66.28 ± 19.33	64.87 ± 16.1	59.86 ± 15.82	50.29 ± 10.86	0.01
Sex male, N (%)	71 (58.2)	27 (71.1)	27 (61.4)	6 (85.7)	0.229
BMI Kg/m^2^, mean ± SD	27.31 ± 5.01	28.52 ± 4.72	27.65 ± 6.47	28.17 ± 8.91	0.506
*Previous diseases, N (%)*					
Diabetes	29 (23.8)	10 (26.3)	10 (22.7)	3 (42.9)	0.627
Higher blood pressure	68 (55.7)	27 (71.1)	20 (45.5)	5 (71.4)	0.807
Cardiovascular disease	31 (25.4)	15 (39.5)	7 (15.9)	0	0.168
Lung disease	19 (15.6)	5 (13.2)	5 (11.4)	3 (42.9)	0.719
Chronic kidney disease	12 (9.8)	7 (18.4)	2 (4.5)	0	0.36
Dyslipidemia	38 (31.1)	15 (39.5)	17 (38.6)	3 (42.9)	0.257
*Pancreatitis etiology, N (%)*					
Biliary	62 (50.8)	26 (68.4)	17 (38.6)	2 (28.6)	0.197
Alcoholic	20 (16.4)	4 (10.5)	14 (31.8)	4 (57.1)	0.005
Idiopathic	25 (20.5)	5 (13.2)	10 (22.7)	0	0.581
Post-ERCP	3 (2.5)	1 (2.6)	1 (2.3)	1 (14.3)	0.408
Other	12 (9.8)	2 (5.3)	2 (4.5)	0	0.142

*TG* triglyceride, *BMI* body mass index, *ERCP* endoscopic retrograde cholangiopancreatography, *Other* drugs, pancreas divisum, autoimmune, intraductal papillary mucinous neoplasm, post-surgical procedure, *SD* standard deviation

### Pancreatic necrosis

**Fig. 2:**
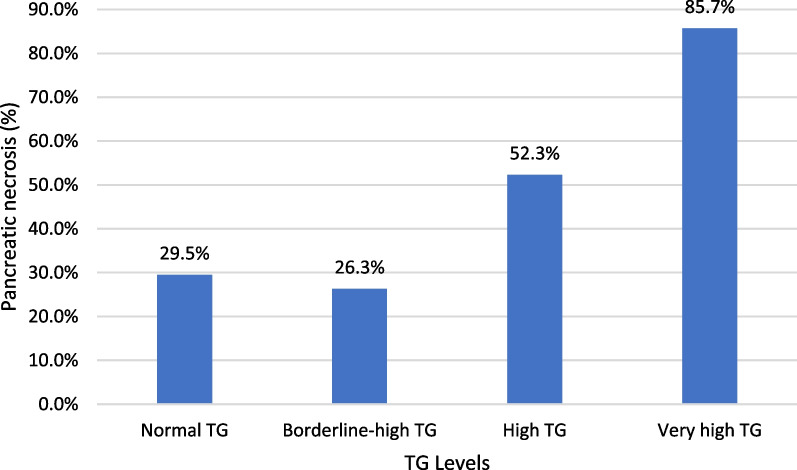
The proportion of pancreatic necrosis for the categories of TG levels in patients with acute pancreatitis. Cochran-Armitage test for trend was significant (*p* = 0.001). TG: triglyceride

Pancreatic necrosis occurred in 35.5% of our population, distributing 29.5% of the patients in the Normal TG group, 26.3% in the Borderline-high TG group, 52.3% in the High TG group, and 85.7% in the Very high TG group (*p* = 0.001) (Table 2). The trend analysis of the proportions between the groups stratified by TG level using the Cochran-Armitage trend test observed a significant association between higher TG levels and the incidence of pancreatic necrosis (*p* = 0.001). (Fig. 2).

**Table 2:** Pancreatic necrosis and complications of acute pancreatitis according to TG levels group

	Normal TG (< 150 mg/dL) N = 122	Borderline-high TG (150–199 mg/dL) N = 38	High TG (200–499 mg/dL) N = 44	Very high TG (≥ 500 mg/dL) N = 7	*p* value
*Local complications, N (%)*					
Fluid collections	50 (41)	14 (36.8)	27 (61.4)	6 (85.7)	0.005
Pancreatic necrosis	36 (29.5)	10 (26.3)	23 (52.3)	6 (85.7)	0.001
Percentage of necrosis < 30%	26 (21.3)	4 (10.5)	12 (27.3)	3 (42.9)	0.298
Percentage of necrosis 30–50%	6 (4.9)	4 (10.5)	4 (9.1)	2 (28.6)	0.051
Percentage of necrosis > 50%	4 (3.3)	2 (5.3)	7 (15.9)	1 (14.3)	0.005
Pancreatic necrosis infection	13 (10.7)	2 (5.3)	6 (13.6)	3 (42.9)	0.135
*Invasive procedure against necrosis, N (%)*					
All types of procedures	14 (11.5)	2 (5.3)	12 (27.3)	3 (42.9)	0.004
Radiological	7 (5.7)	1 (2.6)	7 (15.9)	0	0.181
Endoscopic	7 (5.7)	1 (2.6)	6 (13.6)	1 (14.3)	0.103
Surgical	6 (4.9)	1 (2.6)	1 (2.3)	1 (14.3)	0.92
*Radiological score*					
CTSI, mean ± SD	2.97 ± 2.22	3.03 ± 2.71	4.56 ± 2.79	6.14 ± 2.61	< 0.001
MCTSI, mean ± SD	4.69 ± 2.77	4.41 ± 3.19	5.93 ± 3.08	7.71 ± 2.69	0.007

*TG* triglyceride, *SD* standard deviation, *CTSI* computed tomography severity index, *MCTSI* Modified computed tomography severity index

When we analyze the incidence of systemic complications according to the extent of pancreatic necrosis (< 30%, 30–50% and > 50%), we observe that a greater extent of necrosis presented a higher incidence of organ failure (24.4% vs. 50% vs. 85.7%, *p* < 0.001), severe pancreatitis (15.6% vs. 37.5% vs. 71.4%, *p* < 0.001) and mortality (2.2% vs. 31.2% vs. 35.7%, *p* < 0.001).

### Risk factors for pancreatic necrosis (univariable and multivariable analysis)

Demographic characteristics, comorbidities, etiology of AP, TG levels, and other biochemical markers according to pancreatic necrosis were summarized in Table 3.

**Table 3:** Characteristics of acute pancreatitis according to pancreatic necrosis

	No Pancreatic necrosis	Pancreatic necrosis	*p value*
	N = 136	N = 75	
Age, mean ± SD	64.53 ± 19.13	63.48 ± 16.24	0.414
Sex male, N (%)	78 (57.4)	53 (70.7)	0.056
BMI (Kg/m^2^), mean ± SD	27.62 ± 5.73	27.63 ± 4.77	0.883
*Previous diseases, N (%)*			
Diabetes	34 (25)	18 (24)	0.872
Higher blood pressure	73 (53.7)	47 (62.7)	0.207
Cardiovascular disease	39 (28.7)	14 (18.7)	0.109
Lung disease	18 (13.2)	14 (18.7)	0.292
Chronic kidney disease	18 (13.2)	3 (4)	0.032
Dyslipidemia	46 (33.8)	27 (36)	0.75
*Pancreatitis etiology, N (%)*			
Biliary	64 (47.1)	43 (57.3)	0.153
Alcoholic	27 (19.9)	15 (20)	0.98
Idiopathic	26 (19.1)	14 (18.7)	0.936
Post-ERCP	3 (2.2)	3 (4)	0.453
Other	16 (11.8)	0	0.003
*Serum TG (mg/dL), mean* ± *SD*	144.53 ± 85.81	209.17 ± 181.29	0.015
*TG Category, N (%):*			
(a) Normal TG (< 150 mg/dL)	86 (63.2)	36 (48)	0.032
(b) Borderline-high TG (150–199 mg/dL)	28 (20.6)	10 (13.3)	0.189
(c) High TG (200–499 mg/dL)	21 (15.4)	23 (30.7)	0.009
(d) Very high TG (≥ 500 mg/dL)	1 (0.7)	6 (8)	0.005
TG ≥ 150 mg/dL (b + c + d)	50 (36.8)	39 (52)	0.032
TG ≥ 200 mg/dL (c + d)	22 (16.2)	29 (38.7)	< 0.001
*Biochemical markers at admission*			
Creatinine (mg/dL), mean ± SD	1.05 ± 0.61	1.19 ± 0.58	0.008
Hematocrit (%), mean ± SD	41.28 ± 5.73	44.35 ± 6.37	0.001
BUN (mg/dL), mean ± SD	20.89 ± 11.12	24.55 ± 15.27	0.151
C-reactive protein (mg/dL), mean ± SD	5.99 ± 8.99	9.17 ± 11.08	0.066

*TG* triglyceride, *SD* standard deviation, *BMI* body mass index, *ERCP* endoscopic retrograde cholangiopancreatography, *Other* drugs, pancreas divisum, autoimmune, intraductal papillary mucinous neoplasm, post-surgical procedure, *BUN* Blood urea nitrogen

Multivariable analysis showed that elevated TG levels ≥ 200 mg/dL were associated with the development of pancreatic necrosis (OR: 3.27, 95% CI 1.7–6.27, *p* < 0.001). Hematocrit at admission was also associated with the development of pancreatic necrosis in the multivariable analysis (Table 4).

**Table 4:** Univariate and multivariable analysis of factors associated with pancreatic necrosis

	Univariate analysis		Multivariable analysis	
	OR (95% CI)	*p value*	OR (95% CI)	*p value*
Age ≥ 65 years	0.81 (0.46–1.41)	0.454		
Sex male	1.79 (0.98–3.27)	0.058		
BMI ≥ 30 Kg/m^2^	0.8 (0.42–1.53)	0.507		
Diabetes	0.95 (0.49–1.83)	0.872		
Cardiovascular disease	0.57 (0.29–2.69)	0.111		
Lung disease	1.51 (0.7–3.23)	0.295		
Chronic kidney disease	0.27 (0.08–0.96)	0.043	0.29 (0.07–1.16)	0.08
Dyslipidemia	1.1 (0.61–1.99)	0.75		
Biliary etiology	1.51 (0.86–2.67)	0.154		
Alcoholic etiology	1.01 (0.49–2.04)	0.98		
Triglyceride ≥ 200 mg/dL*	3.27 (1.7–6.27)	< 0.001	3.99 (1.86–8.58)	< 0.001
Creatinine ≥ 1.8 mg/dL	1.01 (0.33–3.15)	0.98		
Hematocrit ≥ 44%	3.29 (1.82–5.98)	< 0.001	4.92 (2.45–9.91)	< 0.001
BUN ≥ 20 mg/dL	1.39 (0.79–2.46)	0.254		
C-reactive protein ≥ 15 mg/dL	2.37 (1.1–5.1)	0.027	1.39 (0.99–1.94)	0.053

*OR* odds ratio, *CI* confidence interval, *BMI* body mass index, *BUN* Blood urea nitrogen

*High TG + Very high TG

### ROC analysis for TG levels and other biochemical markers predicting pancreatic necrosis

Area under the curve (AUC) analysis of TG and biochemical markers predicting pancreatic necrosis were as follows: TG levels: AUC: 0.601 (CI 95% 0.519–0.684), creatinine: AUC: 0.611 (CI 95% 0.53–0.692), and hematocrit at admission AUC ROC: 0.644 (CI 95%, 0.562–0.727). We did not find significant statistical differences when comparing the ROC curves using the Delong test (*p* = 0.709). The ROC curves of the biochemical markers were plotted in the Additional file 1.

### Systemic complications

Incidence of organ failure, multi-organ failure, and persistent organ failure increased significantly and accordingly to the increase in TG levels groups (*p* = 0.009, *p* < 0.001, *p* < 0.001, respectively), but not for mortality (*p* = 0.062).

## Discussion

Our study found that TG levels ≥ 200 mg/dL (High TG and Very high TG) were a risk factor for developing pancreatic necrosis. Elevated TG levels were associated with a higher incidence of pancreatic necrosis, and this association was more significant at higher TG levels.

This study is one of the few studies published in the literature that demonstrated the relation between elevated TG levels and pancreatic necrosis development. In this line, Tariq et al. [41] found that local complications such as pancreatic necrosis are associated with higher TG levels (3.11% vs. 12% in the group of TG > 200 mg/dl,* p* = 0.001).

We also found that higher levels of TG are associated with the extent of parenchymal necrosis. Those findings support our hypothesis that TG has a role in necrosis development and are similar to those reported by Cheng et al. [24]. Another relevant finding was a higher proportion of the need for invasive procedures against necrosis in the elevated triglycerides groups, which agrees with the fact that the extent of pancreatic necrosis is associated with the need for more invasive procedures [42, 43]. However, we did not find a significant association with higher TG values regarding infected pancreatic necrosis. This could be explained because the etiology of infected pancreatic necrosis involves other mechanisms, such as bacterial translocation from the intestinal tract, administration of total parenteral nutrition, and extrapancreatic sources of infections [44–46].

When exploring the role of other potential factors related to necrosis, such as age, sex, and previous comorbidities were not associated with the development of pancreatic necrosis. These results agree with previous studies [37, 38, 47]. In that line, we tested serum creatinine, BUN, hemoconcentration at admission, and C-reactive protein, all previously described as predictors of severity in AP [40, 48]. We found that hemoconcentration at admission was associated with pancreatic necrosis, consistent with previous studies' results [38, 49].

One of the hypotheses explaining the increase of TG in AP is the lipolysis of the visceral fat occurring in the early phases of the disease, considering TGs compose 80% to 90% of the volume of adipocytes [50, 51]. The release of activated pancreatic enzymes (pancreatic lipases), catecholamines, and glucagon into the systemic circulation leads to an accelerated breakdown of adipose tissue, TG releasing, and an increased serum lipid concentration [24, 52, 53]. The increase in TG levels leads to an increase in blood viscosity that further favors microcirculation disorders of the pancreatic parenchyma. In addition, TG can be hydrolyzed by lipases released during pancreatitis [54], and large amounts of free fatty acids (FFA) produced directly damage pancreatic acinar cells and increase the extent of parenchymal necrosis [55, 56]. Also, the excess FFA in the circulation induces positive regulation of cytokines and activation of inflammatory cascades predisposing to organ failure [57].

Our study included a period in which the COVID-19 pandemic occurred. Patients included after inclusion/exclusion criteria did not present active SARS-CoV-2 infection and were not vaccinated in the days before admission for acute pancreatitis. Some authors have reported acute pancreatitis and HTG after COVID-19 vaccination [58, 59].

A recent meta-analysis found that pancreatic necrosis occurs more frequently in alcoholic pancreatitis than in biliary pancreatitis. However, differences in the proportion of pancreatic necrosis by etiology were analyzed in few studies, which does not allow comparison with other etiologies [60]. Our study found no association between alcoholic pancreatitis and pancreatic necrosis.

Our study had limitations, such as not knowing the serum TG levels before the pancreatitis episode. Therefore, it is unclear whether elevated TG levels preceded the development of acute pancreatitis or whether acute pancreatitis caused elevated TG levels. Because CT scans were performed at the discretion of treating physicians, not all patients from the initial cohort underwent CT scan. Our analysis only included patients with CT to avoid this bias. We analyzed the entire initial cohort and the patients who underwent CT scan and found no differences in baseline characteristics.

However, our study has strengths, such as the prospective data collection, the exclusion of HTG-induced AP, and the exclusion of patients with more than 72 h from the onset of symptoms and admission to reduce the bias of including patients with prolonged disease.

We propose considering hypertriglyceridemia as a potential risk factor for pancreatic necrosis development. In that line, it is necessary identified the pathological mechanisms of TG increasing in AP, and the pathways by which TGs and FFA are involved in pancreatic tissue damage and systemic complications, to develop new treatment strategies for diminishing the impact of pancreatic necrosis. Studies in HTG-induced AP suggest that enzyme blockers, early removal of TG, and toxic free fatty acids by plasmapheresis may be advantageous [61–63]; however, there is a lack of studies in patients with AP not induced by HTG.

## Conclusions

Elevated TG levels in the early stages of AP were a risk factor associated with the development of pancreatic necrosis. The incidence of pancreatic necrosis increases proportionally with the severity of HTG. More research is necessary to know the pathophysiological mechanism that explains this relationship and design novel interventions for pancreatic necrosis.

## Supplementary Information

**Additional file 1: Table S1**. Performance of biochemical markers at admission in predicting pancreatic necrosis: Triglyceride ≥ 200 mg/dL, Creatinine ≥ 1.8 mg/dL, Hematocrit ≥ 44%, BUN ≥ 20 mg/dL, C-reactive protein ≥15 mg/dL. **Fig. S1**. Receiver operating characteristic (ROC) curve for pancreatic necrosis of triglycerides and biochemical markers at admission.

## Data Availability

The datasets used and analyzed during the current study are available from the corresponding author on reasonable request.

## References

[1] Fagenholz PJ, Castillo CF, Harris NS, Pelletier AJ, Camargo CA (2007). Increasing United States hospital admissions for acute pancreatitis, 1988–2003. Ann Epidemiol.

[2] Wang S, Li S, Feng Q, Feng X, Xu L, Zhao Q (2011). Overweight is an additional prognostic factor in acute pancreatitis: a meta-analysis. Pancreatology.

[3] Bradley EL (1993). A clinically based classification system for acute pancreatitis summary of the international symposium on Acute Pancreatitis, Atlanta, Ga, September 11 through 13, 1992. Arch Surg.

[4] Singh VK, Bollen TL, Wu BU, Repas K, Maurer R, Yu S (2011). An assessment of the severity of interstitial pancreatitis. Clin Gastroenterol Hepatol.

[5] Banks PA, Freeman ML (2006). Practice Parameters Committee of the American College of Gastroenterology. Practice guidelines in acute pancreatitis. Am J Gastroenterol.

[6] van Santvoort HC, Bakker OJ, Bollen TL, Besselink MG, Ahmed Ali U, Schrijver AM (2011). A conservative and minimally invasive approach to necrotizing pancreatitis improves outcome. Gastroenterology.

[7] Rashid MU, Hussain I, Jehanzeb S, Ullah W, Ali S, Jain AG (2019). Pancreatic necrosis: complications and changing trend of treatment. World J Gastrointest Surg.

[8] Chua TY, Walsh RM, Baker ME, Stevens T (2017). Necrotizing pancreatitis: diagnose, treat, consult. Cleve Clin J Med.

[9] Baron TH, DiMaio CJ, Wang AY, Morgan KA (2020). American gastroenterological association clinical practice update: management of pancreatic necrosis. Gastroenterology.

[10] Havel RJ (1969). Pathogenesis, differentiation and management of hypertriglyceridemia. Adv Intern Med.

[11] Valdivielso P, Ramírez-Bueno A, Ewald N (2014). Current knowledge of hypertriglyceridemic pancreatitis. Eur J Intern Med.

[12] Weinberg JM (2006). Lipotoxicity. Kidney Int.

[13] Das SLM, Singh PP, Phillips ARJ, Murphy R, Windsor JA, Petrov MS (2014). Newly diagnosed diabetes mellitus after acute pancreatitis: a systematic review and meta-analysis. Gut.

[14] Ewald N, Bretzel RG (2013). Diabetes mellitus secondary to pancreatic diseases (Type 3c)–are we neglecting an important disease?. Eur J Intern Med.

[15] Zechner D, Spitzner M, Bobrowski A, Knapp N, Kuhla A, Vollmar B (2012). Diabetes aggravates acute pancreatitis and inhibits pancreas regeneration in mice. Diabetologia.

[16] Mankad P, James A, Siriwardena AK, Elliott AC, Bruce JIE (2012). Insulin protects pancreatic acinar cells from cytosolic calcium overload and inhibition of plasma membrane calcium pump. J Biol Chem.

[17] Samad A, James A, Wong J, Mankad P, Whitehouse J, Patel W (2014). Insulin protects pancreatic acinar cells from palmitoleic acid-induced cellular injury. J Biol Chem.

[18] Anderson F, Thomson SR, Clarke DL, Buccimazza I (2009). Dyslipidaemic pancreatitis clinical assessment and analysis of disease severity and outcomes. Pancreatology.

[19] Deng L-H, Xue P, Xia Q, Yang X-N, Wan M-H (2008). Effect of admission hypertriglyceridemia on the episodes of severe acute pancreatitis. World J Gastroenterol.

[20] Nawaz H, Koutroumpakis E, Easler J, Slivka A, Whitcomb DC, Singh VP (2015). Elevated serum triglycerides are independently associated with persistent organ failure in acute pancreatitis. Am J Gastroenterol.

[21] Lloret Linares C, Pelletier AL, Czernichow S, Vergnaud AC, Bonnefont-Rousselot D, Levy P (2008). Acute pancreatitis in a cohort of 129 patients referred for severe hypertriglyceridemia. Pancreas.

[22] Hidalgo NJ, Pando E, Alberti P, Vidal L, Mata R, Fernandez N (2022). Elevated serum triglyceride levels in acute pancreatitis: a parameter to be measured and considered early. World J Surg.

[23] Lankisch PG, Pflichthofer D, Lehnick D (2000). No strict correlation between necrosis and organ failure in acute pancreatitis. Pancreas.

[24] Cheng L, Luo Z, Xiang K, Ren J, Huang Z, Tang L (2015). Clinical significance of serum triglyceride elevation at early stage of acute biliary pancreatitis. BMC Gastroenterol.

[25] Zeng Y, Wang X, Zhang W, Wu K, Ma J (2012). Hypertriglyceridemia aggravates ER stress and pathogenesis of acute pancreatitis. Hepatogastroenterology.

[26] Banks PA, Bollen TL, Dervenis C, Gooszen HG, Johnson CD, Sarr MG (2013). Classification of acute pancreatitis–2012: revision of the Atlanta classification and definitions by international consensus. Gut.

[27] de Pretis N, Amodio A, Frulloni L (2018). Hypertriglyceridemic pancreatitis: epidemiology, pathophysiology and clinical management. United Eur Gastroenterol J.

[28] Leppäniemi A, Tolonen M, Tarasconi A, Segovia-Lohse H, Gamberini E, Kirkpatrick AW (2019). 2019 WSES guidelines for the management of severe acute pancreatitis. World J Emerg Surg.

[29] Fortson MR, Freedman SN, Webster PD (1995). Clinical assessment of hyperlipidemic pancreatitis. Am J Gastroenterol.

[30] Working Group IAP/APA Acute Pancreatitis Guidelines (2013). IAP/APA evidence-based guidelines for the management of acute pancreatitis. Pancreatology.

[31] National Cholesterol Education Program (NCEP) (2002). Expert panel on detection E and T of HBC in A (Adult TPI. Third Report of the National Cholesterol Education Program (NCEP) expert panel on detection, evaluation, and treatment of high blood cholesterol in adults (adult treatment panel III) final report. Circulation.

[32] Trikudanathan G, Wolbrink DRJ, van Santvoort HC, Mallery S, Freeman M, Besselink MG (2019). Current concepts in severe acute and necrotizing pancreatitis: an evidence-based approach. Gastroenterology.

[33] Balthazar EJ, Robinson DL, Megibow AJ, Ranson JH (1990). Acute pancreatitis: value of CT in establishing prognosis. Radiology.

[34] Mortele KJ, Wiesner W, Intriere L, Shankar S, Zou KH, Kalantari BN (2004). A modified CT severity index for evaluating acute pancreatitis: improved correlation with patient outcome. AJR Am J Roentgenol.

[35] Alberti P, Pando E, Mata R, Vidal L, Roson N, Mast R (2021). Evaluation of the modified computed tomography severity index (MCTSI) and computed tomography severity index (CTSI) in predicting severity and clinical outcomes in acute pancreatitis. J Dig Dis.

[36] Marshall JC, Cook DJ, Christou N (1995). Multiple organ dysfunction score: a reliable descriptor of a complex clinical outcome. Crit Care Med.

[37] Muddana V, Whitcomb DC, Khalid A, Slivka A, Papachristou GI (2009). Elevated serum creatinine as a marker of pancreatic necrosis in acute pancreatitis. Am J Gastroenterol.

[38] Brown A, Orav J, Banks PA (2000). Hemoconcentration is an early marker for organ failure and necrotizing pancreatitis. Pancreas.

[39] Wu BU, Bakker OJ, Papachristou GI, Besselink MG, Repas K, van Santvoort HC (2011). Blood urea nitrogen in the early assessment of acute pancreatitis: an international validation study. Arch Intern Med.

[40] Khanna AK, Meher S, Prakash S, Tiwary SK, Singh U, Srivastava A (2013). Comparison of Ranson, Glasgow, MOSS, SIRS, BISAP, APACHE-II, CTSI Scores, IL-6, CRP, and procalcitonin in predicting severity, organ failure, pancreatic necrosis, and mortality in acute pancreatitis. HPB Surg.

[41] Tariq H, Gaduputi V, Peralta R, Abbas N, Nayudu SK, Thet P (2015). Serum triglyceride level - a predictor of complications and outcomes in acute pancreatitis?. Can J Gastroenterol Hepatol.

[42] Chandrasekhara V, Elhanafi S, Storm AC, Takahashi N, Lee NJ, Levy MJ (2021). Predicting the need for step-up therapy after EUS-guided drainage of pancreatic fluid collections with Lumen-apposing metal stents. Clin Gastroenterol Hepatol.

[43] Cao X, Cao F, Li A, Gao X, Wang X-H, Liu D-G (2017). Predictive factors of pancreatic necrosectomy following percutaneous catheter drainage as a primary treatment of patients with infected necrotizing pancreatitis. Exp Ther Med.

[44] Pando E, Alberti P, Hidalgo J, Vidal L, Dopazo C, Caralt M (2018). The role of extra-pancreatic infections in the prediction of severity and local complications in acute pancreatitis. Pancreatology.

[45] Isenmann R, Beger HG (2001). Bacterial infection of pancreatic necrosis: role of bacterial translocation, impact of antibiotic treatment. Pancreatology.

[46] Petrov MS, Kukosh MV, Emelyanov NV (2006). A randomized controlled trial of enteral versus parenteral feeding in patients with predicted severe acute pancreatitis shows a significant reduction in mortality and in infected pancreatic complications with total enteral nutrition. Dig Surg.

[47] Baillargeon JD, Orav J, Ramagopal V, Tenner SM, Banks PA (1998). Hemoconcentration as an early risk factor for necrotizing pancreatitis. Am J Gastroenterol.

[48] Uhl W, Büchler M, Malfertheiner P, Martini M, Beger HG (1991). PMN-elastase in comparison with CRP, antiproteases, and LDH as indicators of necrosis in human acute pancreatitis. Pancreas.

[49] Koutroumpakis E, Wu BU, Bakker OJ, Dudekula A, Singh VK, Besselink MG (2015). Admission hematocrit and rise in blood urea nitrogen at 24 h outperform other laboratory markers in predicting persistent organ failure and pancreatic necrosis in acute pancreatitis: a post hoc analysis of three large prospective databases. Am J Gastroenterol.

[50] Ren J, Dimitrov I, Sherry AD, Malloy CR (2008). Composition of adipose tissue and marrow fat in humans by 1H NMR at 7 Tesla. J Lipid Res.

[51] Garaulet M, Hernandez-Morante JJ, Lujan J, Tebar FJ, Zamora S (2006). Relationship between fat cell size and number and fatty acid composition in adipose tissue from different fat depots in overweight/obese humans. Int J Obes (Lond).

[52] Murad MH, Hazem A, Coto-Yglesias F, Dzyubak S, Gupta S, Bancos I (2012). The association of hypertriglyceridemia with cardiovascular events and pancreatitis: a systematic review and meta-analysis. BMC Endocr Disord.

[53] Brunzell JD, Schrott HG (2012). The interaction of familial and secondary causes of hypertriglyceridemia: role in pancreatitis. J Clin Lipidol.

[54] Patel K, Trivedi RN, Durgampudi C, Noel P, Cline RA, DeLany JP (2015). Lipolysis of visceral adipocyte triglyceride by pancreatic lipases converts mild acute pancreatitis to severe pancreatitis independent of necrosis and inflammation. Am J Pathol.

[55] Kota SK, Krishna SVS, Lakhtakia S, Modi KD (2013). Metabolic pancreatitis: etiopathogenesis and management. Indian J Endocrinol Metab.

[56] Yang F, Wang Y, Sternfeld L, Rodriguez JA, Ross C, Hayden MR (2009). The role of free fatty acids, pancreatic lipase and Ca+ signalling in injury of isolated acinar cells and pancreatitis model in lipoprotein lipase-deficient mice. Acta Physiol (Oxf).

[57] Navina S, Acharya C, DeLany JP, Orlichenko LS, Baty CJ, Shiva SS (2011). Lipotoxicity causes multisystem organ failure and exacerbates acute pancreatitis in obesity. Sci Transl Med.

[58] Ozaka S, Kodera T, Ariki S, Kobayashi T, Murakami K (2022). Acute pancreatitis soon after COVID-19 vaccination: a case report. Medicine.

[59] Cheung B, Hwang J, Stolarczyk A, Mahlof EN, Block RC (2021). Case study of hypertriglyceridemia from COVID-19 Pfizer-BioNTech vaccination in a patient with familial hypercholesteremia. Eur Rev Med Pharmacol Sci.

[60] Bálint ER, Fűr G, Kiss L, Németh DI, Soós A, Hegyi P (2020). Assessment of the course of acute pancreatitis in the light of aetiology: a systematic review and meta-analysis. Sci Rep.

[61] Jeong YK, Kim H (2017). A mini-review on the effect of docosahexaenoic acid (DHA) on cerulein-induced and hypertriglyceridemic acute pancreatitis. Int J Mol Sci.

[62] Click B, Ketchum AM, Turner R, Whitcomb DC, Papachristou GI, Yadav D (2015). The role of apheresis in hypertriglyceridemia-induced acute pancreatitis: a systematic review. Pancreatology.

[63] Kuchay MS, Farooqui KJ, Bano T, Khandelwal M, Gill H, Mithal A (2017). Heparin and insulin in the management of hypertriglyceridemia-associated pancreatitis: case series and literature review. Arch Endocrinol Metab.

