# Supplementary material for: The role of high serum triglyceride levels on pancreatic necrosis development and related complications
Source: BMC Gastroenterol. 2023 Feb 24;23:51. doi: 10.1186/s12876-023-02684-9 (PMC9955530; doi:10.1186/s12876-023-02684-9)
Supplement: Supplementary file 1 — Additional file 1: Table S1. Performance of biochemical markers at admission in predicting pancreatic necrosis: Triglyceride ≥ 200 mg/dL, Creatinine ≥ 1.8 mg/dL, Hematocrit ≥ 44%, BUN ≥ 20 mg/dL, C-reactive protein ≥15 mg/dL. Fig. S1. Receiver operating characteristic (ROC) curve for pancreatic necrosis of triglycerides and biochemical markers at admission. [file 12876_2023_2684_MOESM1_ESM.docx]

**Supplementary material**

Table 1. Performance of biochemical markers at admission in predicting pancreatic necrosis: Triglyceride ≥200mg/dL, Creatinine ≥1.8 mg/dL, Hematocrit ≥44%, BUN ≥20mg/dL, C-reactive protein ≥15mg/dL.

|  | AUC (CI 95%) | P | Sensitivity | Speciﬁcity | PPV | NPV |
| --- | --- | --- | --- | --- | --- | --- |
| **Biochemical markers** |  |  |  |  |  |  |
| Triglyceride | 0.601 (0.519-0.684) | 0.015 | 38.7% | 83.8% | 56.9% | 71.3% |
| Creatinine | 0.611 (0.53-0.692) | 0.008 | 6.8% | 93.3% | 35.7% | 64.6% |
| Hematocrit | 0.644 (0.562-0.727) | 0.001 | 53.3% | 74.3% | 53.3% | 74.3% |
| BUN | 0.56 (0.477-0.643) | 0.152 | 52.7% | 55.6% | 39.4% | 68.2% |
| C-reactive protein | 0.579 (0.494-0.663) | 0.066 | 23.9% | 88.3% | 53.1% | 67.7% |

*AUC: area under the curve, BUN:* Blood urea nitrogen.


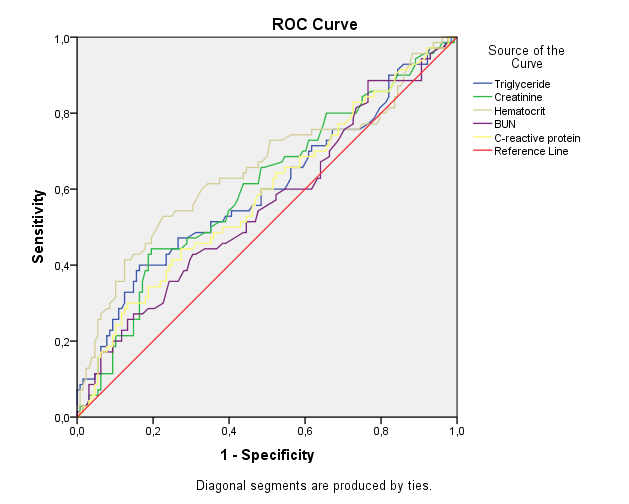


Figure 1. Receiver operating characteristic (ROC) curve for pancreatic necrosis of triglycerides and biochemical markers at admission.
